# Supplementary material for: Tensile Strength Essay Comparing Three Different Platelet-Rich Fibrin Membranes (L-PRF, A-PRF, and A-PRF+): A Mechanical and Structural In Vitro Evaluation
Source: Polymers (Basel). 2022 Mar 29;14(7):1392. doi: 10.3390/polym14071392 (PMC9002533; doi:10.3390/polym14071392)
Supplement: Supplementary file 1 [file polymers-14-01392-s001.zip › polymers-1635659-supplementary.pdf]

Supplementary Materials

Table S1. Essential platelet growth factor properties.

Growth Factors	Biological Function
PDGF	• Stimulates mitogenic activity and promotes proliferation in mesenchymal cell lines.<br>• Chemotaxis of neutrophils, macrophages, fibroblasts, and other leukocytes but the main target is osteoblasts, which are useful for inducing bone regeneration (Caplan and Correa, 2011).<br>• Collagen synthesis and tissue remodeling by upregulating pathways to break down the old collagen (Jinnin et al., 2005).
VEGF	• Allows the formation and development of new blood vessels through the migration and differentiation of endothelial cells that restore tissue perfusion and microcirculation. The formed vessels are responsible for blood supply to the wound site.<br>• Induce bone tissue formation through the recruitment of macrophages (Hu and Olsen, 2016).
EGF	• Even at low concentrations, this has a higher potential than bFGF to increase osteogenic mineralization in dental pulp stem cells (DPSCs).<br>• Induces the formation of the peri-implant junctional epithelium (Del Angel-Mosqueda et al., 2015).<br>• Pro-inflammatory function that acts as a mechanosensitizer through the production of fibronectin (Müller-Deubert et al., 2017; Teramatsu et al., 2014).
TGF-β1	• Low concentrations are sufficient to induce the proliferation of fibroblasts, osteoblasts, and chondroblasts.<br>• Involved in complex mediation roles such as chemotaxis, mitogenesis, differentiation, apoptosis, remodeling and immunoregulation.<br>• Induces monocyte and neutrophil chemotaxis (Hinz, 2015).<br>• Osteoinduction and progression of osteogenesis (Zhang et al., 2019).
IGF	• Proliferation and differentiation of various types of mesenchymal cells. Its intervention overlaps with that of TGF-beta (Miron et al., 2017).<br>• Synthesis of type I collagen and differentiation of osteoblasts through an important mediator, LARP6, verifying the mineralization capacity (Guo et al., 2017).
HGF	• Regulation of cell morphogenesis.<br>• Matrix deposition and degradation, playing an antifibrotic role that allows the reepithelization of the wound.<br>• Under physiological conditions, interacts with the mesenchymal epithelium (Fukushima et al., 2018).
bFGF	• Involvement in and reinforcement of various proliferation processes of different types of cells such as fibroblasts, mesenchymal stem cells, and osteoprecursor cells.<br>• Stimulates the expression of tissue metalloproteinase inhibitors (TIMP-1) partially decreasing high concentrations of type I collagen, thus hypothesizing its importance in the reorganization of collagen fibers, avoiding excessive deposits with unwanted effects (Chang et al., 2020).<br>• Treatment of periodontal disease, increasing the migration and proliferation of periodontal ligament stem cells (PDLSCs) (Kang et al., 2019).
